# Endoscopic submucosal dissection for diverticulum using combination of countertraction and circumferential-inversion method

**DOI:** 10.1055/a-2239-3468

**Published:** 2024-01-30

**Authors:** Hiroshi Takayama, Yoshinori Morita, Toshitatsu Takao, Douglas Motomura, Madoka Takao, Takashi Toyonaga, Yuzo Kodama

**Affiliations:** 1 592910 Division of Gastroenterology, Kobe University Graduate School of Medicine Department of Internal Medicine, Kobe, Japan; 2 Department of Gastroenterology, Kobe University Hospital International Clinical Cancer Research Center, Kobe, Japan; 3 Department of Gastroenterology, University of British Columbia, Vancouver, Canada

Endoscopic submucosal dissection (ESD) has been attempted in colorectal tumors involving diverticula, which lack a muscularis propria 1 . However, the R0 resection rate in tumors infiltrating the interior of the diverticulum remains low, and there is a risk of perforation 2 3 4 . We recently reported a novel traction method called the circumferential-inversion method, which inverts the lesion circumferentially 5 . Here, we describe the effectiveness of an innovative ESD approach combining countertraction and the circumferential-inversion method for a diverticulum-infiltrating tumor ( Video 1 ).

Endoscopic submucosal dissection using a novel approach that combines the countertraction and circumferential-inversion method for diverticulum-infiltrating tumors.Video 1

The case was an 18-mm 0–IIa tumor involving a diverticulum in the sigmoid colon ( Fig. 1 **a** ). The tumor infiltrated and fully covered a diverticulum in its center ( Fig. 1 **b** ). Following complete circumferential incision and trimming, the specimen was grasped at four points using an 8-mm diameter orthodontic rubber band and clips (SureClip 8 mm; Micro-Tech, Nanjing, China) ( Fig. 1 **c** ). By combining a water pressure method and the circumferential-inversion method, we were able to sufficiently dissect fibrotic submucosa around the central diverticulum. However, dissection of the submucosa inside the diverticulum remained challenging ( Fig. 1 **d** ). Therefore, we fixed the rubber band to the contralateral mucosa using an additional clip for countertraction ( Fig. 2 **a** ). As a result, the tumor inside the diverticulum was pulled into the lumen by the clips, which held the specimen circumferentially ( Fig. 2 **b** ). Additional dissection facilitated complete separation of the tumor from the diverticulum ( Fig. 2 **c** ) and R0 resection was completed without complications ( Fig. 2 **d** ).

**Fig. 1:**
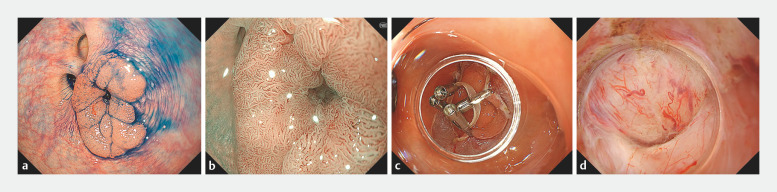
Endoscopic submucosal dissection using the circumferential-inversion method. **a** An 18-mm 0–IIa tumor involving the diverticulum in the sigmoid colon. **b** The tumor infiltrated and fully covered a diverticulum in its center. **c** After a complete circumferential incision and trimming were performed, the specimen was grasped at four points using an 8-mm diameter orthodontic rubber band and clips. **d** By combining a water pressure method and the circumferential-inversion method, we were able to sufficiently dissect fibrotic submucosa around the central diverticulum. However, dissection of the submucosa inside the diverticulum remained challenging.

**Fig. 2:**
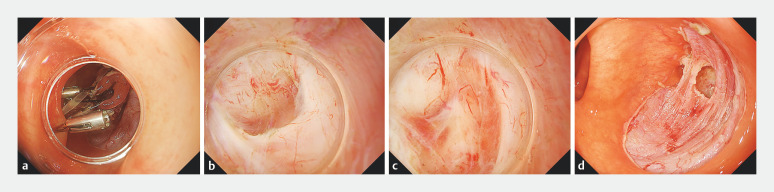
Endoscopic submucosal dissection using a novel approach that combines the countertraction and circumferential-inversion method. **a** We fixed the rubber band to the contralateral mucosa using an additional clip for countertraction. **b** The tumor inside the diverticulum was pulled into the lumen by the clips, which held the specimen circumferentially. **c** Additional dissection facilitated complete separation of the tumor from the diverticulum. **d** R0 resection was completed without complications.

In summary, the circumferential-inversion method is an inversion traction method that simplifies tumor dissection around the central diverticulum from all directions. Additionally, the data reported here demonstrate that the combination of countertraction and the circumferential-inversion method enable successful removal of a tumor from inside the diverticulum in the correct direction. We propose that the circumferential-inversion method facilitates ESD for diverticulum-infiltrating tumors.

Endoscopy_UCTN_Code_TTT_1AO_2AG

## Notes

Endoscopy E-Videos https://eref.thieme.de/e-videos
*E-Videos* is an open access online section of the journal *Endoscopy* , reporting on interesting cases and new techniques in gastroenterological endoscopy. All papers include a high-quality video and are published with a Creative Commons CC-BY license. Endoscopy E-Videos qualify for HINARI discounts and waivers and eligibility is automatically checked during the submission process. We grant 100% waivers to articles whose corresponding authors are based in Group A countries and 50% waivers to those who are based in Group B countries as classified by Research4Life (see: https://www.research4life.org/access/eligibility/ ). This section has its own submission website at https://mc.manuscriptcentral.com/e-videos .
